# Texture analysis of pulmonary parenchymateous changes related to pulmonary thromboembolism in dogs – a novel approach using quantitative methods

**DOI:** 10.1186/s12917-017-1117-1

**Published:** 2017-07-11

**Authors:** C. B. Marschner, M. Kokla, J. M. Amigo, E. A. Rozanski, B. Wiinberg, F. J. McEvoy

**Affiliations:** 1 Department of Veterinary Clinical Sciences, Faculty of Health and Medical Sciences, University of Copenhagen, Groennegaardsvej 3 ground floor, DK-1870 Frederiksberg C, Denmark; 2 Department of Bioinformatics, Faculty of Science, University of Copenhagen, Copenhagen, Denmark; 3 Department of Food Science, Faculty of Science, University of Copenhagen, Copenhagen, Denmark; 4 Department of Clinical Sciences, Cummings School of Veterinary Medicine, Tufts University, Medford, USA; 5 Present address: Institute of Public Health and Clinical Nutrition, University of Eastern Finland, Kuopio campus, Joensuu, Finland; 6 Present address: Global Research Unit, Novo Nordisk A/S, Maaloev, Denmark

**Keywords:** Quantitative analysis, Computed tomography pulmonary angiography, CTPA, Image analysis, Grey level co-occurrence matrix

## Abstract

**Background:**

Diagnosis of pulmonary thromboembolism (PTE) in dogs relies on computed tomography pulmonary angiography (CTPA), but detailed interpretation of CTPA images is demanding for the radiologist and only large vessels may be evaluated. New approaches for better detection of smaller thrombi include dual energy computed tomography (DECT) as well as computer assisted diagnosis (CAD) techniques. The purpose of this study was to investigate the performance of quantitative texture analysis for detecting dogs with PTE using grey-level co-occurrence matrices (GLCM) and multivariate statistical classification analyses.

CT images from healthy (*n* = 6) and diseased (*n* = 29) dogs with and without PTE confirmed on CTPA were segmented so that only tissue with CT numbers between −1024 and −250 Houndsfield Units (HU) was preserved. GLCM analysis and subsequent multivariate classification analyses were performed on texture parameters extracted from these images.

**Results:**

Leave-one-dog-out cross validation and receiver operator characteristic (ROC) showed that the models generated from the texture analysis were able to predict healthy dogs with optimal levels of performance. Partial Least Square Discriminant Analysis (PLS-DA) obtained a sensitivity of 94% and a specificity of 96%, while Support Vector Machines (SVM) yielded a sensitivity of 99% and a specificity of 100%. The models, however, performed worse in classifying the type of disease in the diseased dog group: In diseased dogs with PTE sensitivities were 30% (PLS-DA) and 38% (SVM), and specificities were 80% (PLS-DA) and 89% (SVM). In diseased dogs without PTE the sensitivities of the models were 59% (PLS-DA) and 79% (SVM) and specificities were 79% (PLS-DA) and 82% (SVM).

**Conclusion:**

The results indicate that texture analysis of CTPA images using GLCM is an effective tool for distinguishing healthy from abnormal lung. Furthermore the texture of pulmonary parenchyma in dogs with PTE is altered, when compared to the texture of pulmonary parenchyma of healthy dogs. The models’ poorer performance in classifying dogs within the diseased group, may be related to the low number of dogs compared to texture variables, a lack of balanced number of dogs within each group or a real lack of difference in the texture features among the diseased dogs.

**Electronic supplementary material:**

The online version of this article (doi:10.1186/s12917-017-1117-1) contains supplementary material, which is available to authorized users.

## Background

Computed tomography pulmonary angiography (CTPA) is considered the gold standard for the diagnosis of pulmonary thromboembolism (PTE) in human medicine [1, 2]. In veterinary medicine, no evidence based gold standard for diagnosing PTE exists, but a few studies have shown that CTPA is feasible for the diagnosis of PTE in dogs [3–5].

PTE is identified on CTPA as partial or complete intraluminal filling defects or abrupt cut-off of the pulmonary arteries after contrast injection. The advantages of CTPA include ability to evaluate the entire thorax, thereby allowing an investigation of the various possible causes of respiratory distress which will include PTE but also other pulmonary pathologies such as malignancies or pneumonias [6]. The disadvantages, though, include the radiation dose, use of contrast material, the large amount of data generated after each scan and the need for specialized assessment [7]. Furthermore, since the detection rate for clots decreases with size, clots distal to segmental vessels may be beyond the resolution limits of the technique and go undetected. This issue of resolution limits the use of CTPA for identification of minor clots in the pulmonary circulation [8]. Such small clots may however still be clinically important since they can alter the pulmonary parenchymal perfusion [9]. Based on the limitations of CTPA, it is obvious that tools for better identification of both large and small pulmonary thrombi as well as parenchymateous changes related to these would increase the applicability of CTPA for correct identification of PTE in dogs.

In human medicine perfusion assessment using Dual Energy CT (DECT) is being increasingly investigated since this technique provides morphological as well as functional information derived from one scan, creating a possibility of evaluating not only intravascular changes but also the effects of thrombi on pulmonary parenchyma [10]. However, DECT is not widely available, especially within the veterinary field. An alternative way to assess parenchymal changes due to perfusion defects could be quantitative assessment using computer-based texture analyses. Such computer-assisted diagnosis (CAD) analyses of the pulmonary parenchyma have been investigated in human studies on pulmonary emphysema and small pulmonary nodules with promising results, but not in studies on PTE [11, 12]. One widely used approach in quantitative texture analysis is the application of grey level co-occurrence matrices (GLCM) originally introduced by Haralick in the 1970’s [13, 14]. These matrices record the frequency of occurrence of each possible grey level pairing in a particular image and texture features calculated from the generated matrices reflect the level of heterogeneity in an image. This is calculated in all possible orientations (4 in all) for neighbouring pixels. From these frequency tables a number of different statistical parameters can be estimated [13]. These parameters, which are based on first, second and higher order statistics, collect the spatial variation in the studied image, i.e. the texture of the image; and as such they are essential in differentiating images with different distributions of the grey scale levels.

The objective of the present study was to investigate the performance of quantitative texture analysis using GLCM as a CAD tool for diagnosis of PTE in dogs. We assumed that thrombi induce heterogeneous pulmonary parenchyma texture changes and hypothesized that these changes would enable the discrimination of dogs with macroscopically visible PTE on CTPA from healthy dogs and diseased dogs without PTE on CTPA by means of quantitative texture analysis of the pulmonary parenchyma.

## Methods

The present study was performed at University of Copenhagen utilizing CTPA scans that were generated in a prospective study on the haemostatic and inflammatory changes in dogs suspected of PTE, where CTPA was used to determine presence or absence of emboli. The prospective study was performed in collaboration with Foster Hospital for Small Animals at Cummings School of Veterinary Medicine, Tufts University, North Grafton, MA, USA. Dogs were enrolled and had CTPA performed at Tufts, while the image analyses were performed at University of Copenhagen.

Clinically healthy Beagle dogs from the research colony at Tufts were included as controls prior to inclusion of diseased dogs. Inclusion criteria for the diseased dogs in the prospective study were as follows: Written informed consent granted by the owner, body weight above 10 kg, minimum age of 6 months and clinical signs of respiratory distress or a condition known to predispose to hypercoagulability and/or development of thrombi (immune mediated haemolytic anaemia, sepsis, severe polytrauma, protein losing enteropathy- or nephropathy or systemic neoplasia) were required for inclusion. Dogs with cardiac disease as assessed by a cardiologist were excluded.

### CTPA scanning technique

The CT scans were performed using a 16-row detector helical CT unit (Toshiba Aquilion 16, Toshiba, CA, USA), with tube voltage of 120KV, tube current of 250 mA (small dogs) or 300 mA (for medium and large dogs) and a native resolution of 1 mm. Scans were performed before, during and after injection of intravenous contrast medium. Dogs were under general anaesthesia and placed in sternal recumbency, were hyperventilated and Positive End Expiratory Pressure was applied to minimize risk of atelectasis. Pre-contrast scans were performed using a thorax algorithm and were reconstructed in transverse, sagittal and dorsal planes. Scans were repeated during intravenous injection of Iohexol-300 (600 mg I/kg, diluted to 150 mg I/ml, using a pressure injector with an injection rate of 4 ml/s.) and reconstructed in a soft tissue algorithm in transverse, dorsal and sagittal planes. Post contrast scans obtained 3 min after contrast administration were performed using a lung algorithm and were reconstructed in transverse, dorsal and sagittal planes. Acquisition of slices in pre- and post contrast scans had thickness that varied between 2, 3 or 5 mm according to the patient size. Slice thickness from scans during injection of contrast medium as well as all reconstructed slices were 1 mm. All scans were reviewed regarding presence of pulmonary emboli by board certified radiologists; both at the University of Copenhagen (FJM) and at Tufts (by the duty radiologist on the day of acquisition). Partial or complete intraluminal filling defects visualized on slices obtained during the angiographic phase were used as features identifying PTE in diseased dogs. Healthy dogs were assessed as normal, when absences of pulmonary textural and angiographic changes were confirmed.

### Pre-analysis processing of images

**Fig. 1:**
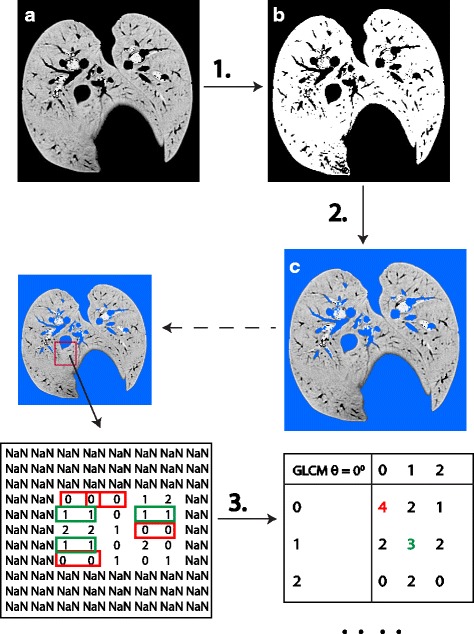
A schematic exemplification of how a grey level co-occurrence matrix is being created in the horizontal direction. Schematic illustration of the pre-analysis processing of images. The original image (**a**) has been transformed to a binary image (**b**) for the separation of the pulmonary parenchyma from the background (step 1). The background of the image is set to NaN (**c**) (step 2) and the image is ready for the estimation of the GLCMs in the horizontal direction (step 3). The *red* and *green rectangles* indicate how often the pixel pairs (0, 0) and (1, 1) are occurring in the image respectively

For each dog, the original transverse DICOM CT images obtained during contrast administration were processed in ImageJ (ImageJ64, 1.43). All tissue between −1024 and −250 HU was preserved in the images, while the rest of the pixels in the images were set to zero. This segmented the images so that only pulmonary parenchyma remained, thus excluding poorly aerated lung (HU-250 to −101), nonaerated lung (−100 to 100 HU) as well as areas of fibrosis, consolidation, some areas pneumonia and any other pathologies with HU greater than −250. This part of the pre-analysis processing was performed by CBM, who was not blinded to the results of the CT interpretation. The segmented images were saved as a tagged TIFF format. Before the calculation of the GLCMs matrices a previous segmentation step was performed. The images were transformed into binary images (Fig. 1 (b)), with one (1) representing the pulmonary parenchyma and zero (0) the background (black color). After the binarization of the images, the background color was set to NaN (Not a Number) and the pulmonary parenchyma intensities received the original values before the binarization (Fig. 1 (c)). This procedure was done in order to separate the background from the foreground (pulmonary parenchyma) so that GLCMs were calculated only for the areas of interest. This part of the pre-analysis processing was performed by MK, who was not blinded to the results of the CT interpretation. Figure 1 illustrates how the images were prepared prior the multivariate analysis by using one image from a healthy dog as an example and a small random segment for the calculation of the GLCMs in the horizontal direction.

### Multivariate statistical classification analyses

Data from GLCMs can be analysed using a number of different statistical methods of which Principal Component Analysis (PCA) [15], Partial Least Square Discriminant Analysis (PLS-DA) [16] and Support Vectors Machines (SVM) [17] are commonly used for different purposes. PCA is used here as a first approach to investigate if any pattern or trend exists in the data by considering the variance of the statistical parameters. PCA is based on projecting the original dataset onto a lesser number of new axes, i.e. principal components (PCs) while maximizing the variance [18].

The main purpose of PLS-DA and SVM-DA methods is for any given classification problem to predict the class of new unknown samples correctly. Both of these algorithms have a supervise nature since a previous calibration (learning) step with well-known samples is needed to generate a model and, then, to predict the unknown category of new samples.

PLS-DA is a linear class modeling algorithm that is based on Partial Least Squares Regression (PLS) and Discriminant Analysis (DA). The PLS regression model relates the independent variables (here textural parameters) to a dependent variable **Y** which contains the class information of the samples. In this way when unknown new samples are introduced into the model, PLS-DA predicts the class that they belong. For a typical two-class classification problem the dependent variable is a vector ***y*** which includes ones (1) and zeros (0) with the same length as the number of samples in the calibration dataset. The number one in ***y*** indicates that the sample belongs to the class of interest and zero indicates that the sample belongs to a different class. In a multiclass classification problem like in the current case (3 classes: Healthy, diseased with PTE and diseased without PTE) *the one* vs *rest strategy* [19, 20] is being used in order to create the dependent variable.

SVM can efficiently perform a linear and a non-linear binary classification. In our analysis we choose an extension version of SVM that can handle multiclass problems [20, 21]. SVM maps the data into a high-dimensional feature space using a Gaussian kernel function. With this approach the samples are divided among the three different classes by constructing a hyperplane or a set of hyperplanes in this high dimensional feature space. When a new sample is introduced it is mapped into the same high dimensional space and predicted to belong to a class based on which side of the hyperplane the sample will fall.

### Data pre-processing and cross-validation setup

The post–contrast CTPA scans were analysed using grey-level co-occurrence matrix analysis [13]. A total of 22 texture features were derived from the image’s GLCMs to characterize the textural properties of the images, such as spatial structure, contrast, roughness, orientation, etc.. An overview of the 22 features is shown in Table 1. For each image four GLCMs were obtained, one for each possible orientation of neighbouring pixels (horizontal, vertical, left diagonal, and right diagonal, corresponding to 0, 45, 90 and 135 degrees respectively) as shown as step 3 in Fig. 1. The calculation of the GLCMs was performed using a resolution of 64 grey scale levels in order to retrieve adequate information from the images [22].

**Table 1:** Overview of the 22 texture features

	First and second order statistical parameters	Abbreviation
1	Autocorrelation [34]	autoc
2	Contrast [13, 34]	contr
3	Correlation (Matlab)	corrm
4	Correlation [13, 34]	corrp
5	Cluster prominence [34]	cprom
6	Dissimilarity [34]	dissi
7	Energy [13, 34]	energ
8	Entropy [34]	entro
9	Inverse difference is homogeneity [22]	homom
10	Homogeneity [34]	homop
11	Maximum probability [34]	maxpr
12	Sum of squares (variance) [13]	sosvh
13	Sum of average [34]	savgh
14	Sum of variance [34]	svarh
15	Sum of entropy [34]	senth
16	Difference variance [34]	dvarh
17	Difference entropy [34]	denth
18	Information measure of correlation 1 [34]	inf1h
19	Information measure of correlation 2 [34]	inf2h
20	Inverse difference normalized [22]	indnc
21	Inverse different moment normalized [22]	indmc
22	Cluster shade [34]	cshad

Overview of and nomenclature for the 22 texture features derived from the grey level co-occurrence matrices generated. One parameter was created in Matlab (noted in brackets)

PLS-DA and SVM models were cross-validated by using a leave-one-dog-out cross validation setup. Briefly, one dog is removed from the training set and a model is generated from the remaining 34 dogs. After that, the classification of the dog that was removed is predicted using the model and parameters of the model’s performance are recorded. This operation is repeated, removing a different dog on each occasion, as many times as dogs in the training set. The overall performance of the classification technique is the average of the performances of the total number of PLS-DA or SVM models constructed.

Image processing and GLCM calculation was performed using the Image processing Toolbox in MATLAB v. 7.0 (The MathWorks Inc., MA, USA). PLS-DA and SVM models were performed using the PLS_Toolbox v. 3.5 (Eigenvector Research Inc., WA, USA) in MATLAB v. 7.0.

### Results

Based on unanimous assessments by the radiologists, the dogs were grouped as healthy (*n* = 6), diseased with PTE (*n* = 7) or diseased without PTE (*n* = 22). The demographics of the included dogs are shown in Table 2.

**Table 2:** Demographics of all included dogs

GROUP	BREED	AGE	SEX
Healthy	Beagle	4 years	M/C
Healthy	Beagle	4 years	M/C
Healthy	Beagle	3 years	F/S
Healthy	Beagle	3 years	F/S
Healthy	Beagle	3 years	F/S
Healthy	Beagle	3 years	F/S
Diseased with PTE	German Shepherd	11 years	M
Diseased with PTE	Australian Shepherd	11 years	F/S
Diseased with PTE	Siberian Husky	7 years	F/S
Diseased with PTE	German Shepherd	3 years	F/S
Diseased with PTE	German Shepherd	1 year	M/C
Diseased with PTE	Viszla	2 years	F/S
Diseased with PTE	German Shepherd	10 years	M
Diseased without PTE	Golden Retriever	11 years	F/S
Diseased without PTE	Cocker Spaniel	7 years	M/C
Diseased without PTE	Silky Terrier	13 years	F/S
Diseased without PTE	English Springer Spaniel	9 years	F/S
Diseased without PTE	Pug	9 years	F/S
Diseased without PTE	English Bulldog	9 years	M/C
Diseased without PTE	Cocker Spaniel	12 years	M/C
Diseased without PTE	Gordon Setter	10 years	F/S
Diseased without PTE	Mixed breed	7 years	M/C
Diseased without PTE	Weimaraner	11 years	F/S
Diseased without PTE	Bernese Mountain Dog	4 years	F/S
Diseased without PTE	Cocker Spaniel	7 years	M/C
Diseased without PTE	Fox Terrier	12 years	F/S
Diseased without PTE	Jack Russel Terrier	8 years	M/C
Diseased without PTE	West Highland White Terrier	12 years	F/S
Diseased without PTE	Mixed breed	10 years	F/S
Diseased without PTE	Labrador Retriever	3 years	F/S
Diseased without PTE	Labrador Retriever	12 years	F/S
Diseased without PTE	Golden Retriever	10 years	F/S
Diseased without PTE	Labrador Retriever	4 years	F/S
Diseased without PTE	Beagle	10 years	F/S
Diseased without PTE	Mixed breed	13 years	M/C

PCA was the first analysis carried out, since this provide a very good first assessment to indicate which, if any, of the variables are important for discrimination between the groups.

**Fig. 2:**
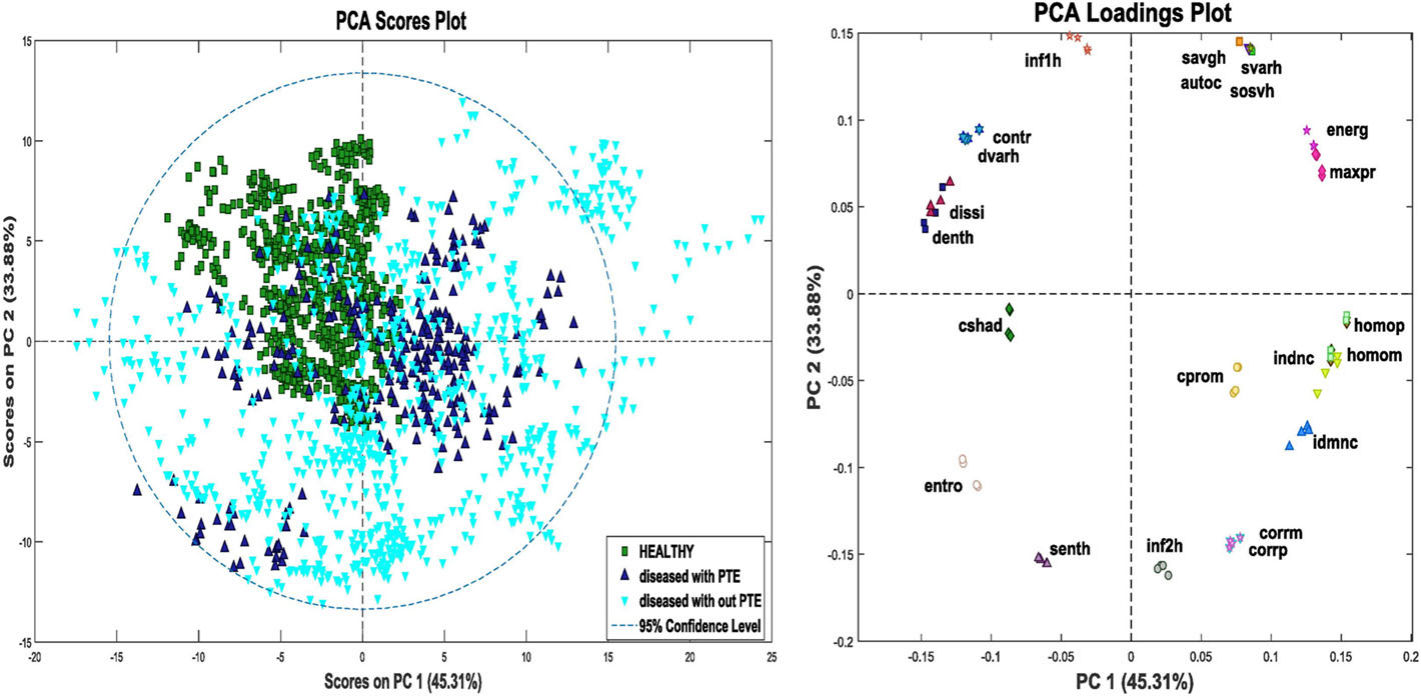
Scores plot and loadings plot from PCA. The plot on the left is the scores plot of samples grouped according to the first two components for PCA when all dogs are used. Each dot represents one CTPA slice from one dog. PCA achieved good separation between slices from the healthy and the diseased dogs, but failed to discriminate between diseased dogs with PTE and diseased dogs without PTE. The plot on the right is the loadings plot of the 22 statistical parameters in four directions for the first two principal components. This plot shows the relationship of the various texture features that were extracted from GLCM and applied in later classification analyses using PLS-DA and SVM. (Nomenclature for these features is given in Table 1)

PCA achieved good separation between slices from healthy and the diseased dogs, but did struggle to discriminate between diseased dogs with PTE and diseased dogs without PTE. However, the results of PCA did indicate that data was suitable for PLS-DA and SVM classification analysis. Scores and loadings plots generated from PCA are shown in Fig. 2.

**Fig. 3:**
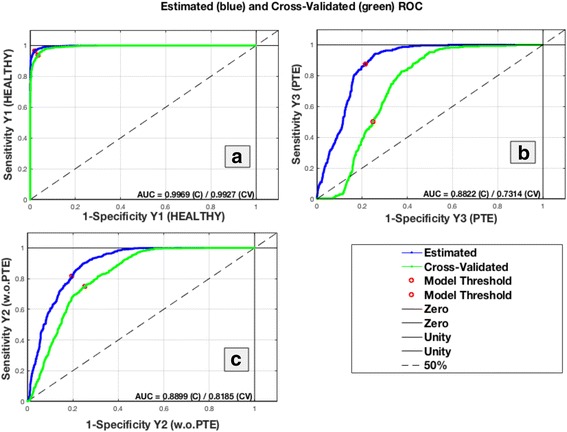
PLS-DA ROC curve. Schematic Illustration of ROC curves of PLS-DA classification on textural features using CTPA slices from 35 dogs. The green line represents the predicted ROC curve whereas the dark blue line represents the leave-one-dog-out cross-validation ROC curve. The plot in (**a**) is a plot of test sensitivity (y coordinate) versus its specificity (x coordinate) for the healthy dogs. The plot in (**b**) is a plot of test sensitivity (y coordinate) versus its specificity (x coordinate) for the diseased dogs with PTE. The plot in (**c**) is a plot of test sensitivity (y coordinate) versus its specificity (x coordinate) for the diseased dogs without PTE

A comparative study of the classification accuracy was therefore performed using PLS-DA and SVM. The ability of these classifiers to correctly assign the data into the three groups can be seen in Table 3. We carried out ROC analysis on the first-order and second-order statistical features to offer a more complete picture of the performance of the PLS-DA classifier. The ROC curves are shown in Fig. 3.

**Table 3:** Sensitivity, specificity, confidence intervals and classification error for the performance of PLS-DA and SVM

	Sensitivity	CI lower bound	CI upper bound	Specificity	CI lower bound	CI upper bound	Classification error
PLSDA							
Healthy (CA)	0,9734	0,9597	0,9825	0,979	0,9687	0,986	0,02335
Healthy (CV)	0,9443	0,9261	0,9583	0,9616	0,9485	0,9715	0,04565
Diseased with PTE (CA)	0,5912	0,5344	0,6457	0,8558	0,8377	0,8722	0,18,577
Diseased with PTE (CV)	0,3041	0,2544	0,3587	0,8029	0,7826	0,8217	0,27,548
Diseased without PTE (CA)	0,7043	0,6717	0,7349	0,884	0,83,636	0,9017	0,19,214
Diseased without PTE (CV)	0,589	0,5545	0,6226	0,7947	0,7696	0,8176	0,29,246
SVM							
Healthy (CA)	1	0,9925	1	1	0,9925	1	0
Healthy (CV)	0,9835	0,9721	0,09904	0,9963	0,9906	0,9986	0,00902
Diseased with PTE (CA)	0,9189	0,8822	0,9449	0,9836	0,9761	0,9888	0,02654
Diseased with PTE (CV)	0,3885	0,3348	0,4451	0,8967	0,8808	0,9107	0,18,312
Diseased without PTE (CA)	0,9674	0,9527	0,9777	0,9779	0,9673	0,9851	0,02654
Diseased without PTE (CV)	0,7895	0,7598	0,8163	0,8214	0,7975	0,843	0,19,214

*CA* Classification, *CV* Cross-validation

The PLS-DA model could successfully discriminate the healthy group from the two diseased groups with a sensitivity of 94% and a specificity of 96% in cross validation, indicating that the texture in the CTPA images of the healthy dogs is different to that seen in the other two groups. PLS-DA performed less well in discriminating between the two diseased groups, with sensitivities of 59% (for dogs without PTE) and 30% (for dogs with PTE) and specificities of 79% (for dogs without PTE) and 80% (for dogs with PTE) in cross-validation, indicating that the texture in the CTPA images of diseased dogs with and without PTE were more difficult to discriminate.

The SVM model yielded excellent sensitivity (99%) and specificity (100%) in cross validation when discriminating between the healthy and the diseased group. However, again lower classification accuracies were generated for discrimination of diseased dogs without PTE (sensitivity 79%, specificity 82%) from dogs with PTE (sensitivity 38%, specificity 89%) in cross validations.

**Fig. 4:**
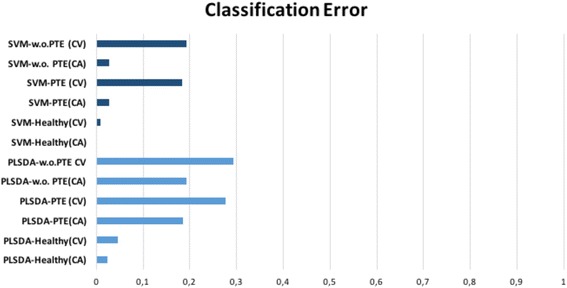
Classification Error Rate between PLS-DA and SVM. Classification errors for PLS-DA (dark blue) and SVM (light blue) for both cross-validation and calibration models

Figure 4 illustrates the Classification Errors of PLS-DA and SVM methods. In our case SVM produced smaller errors while predicting the three classes than PLS-DA, which can be attributed to the non-linear nature (Gaussian kernel) of the classifier.

**Fig. 5:**
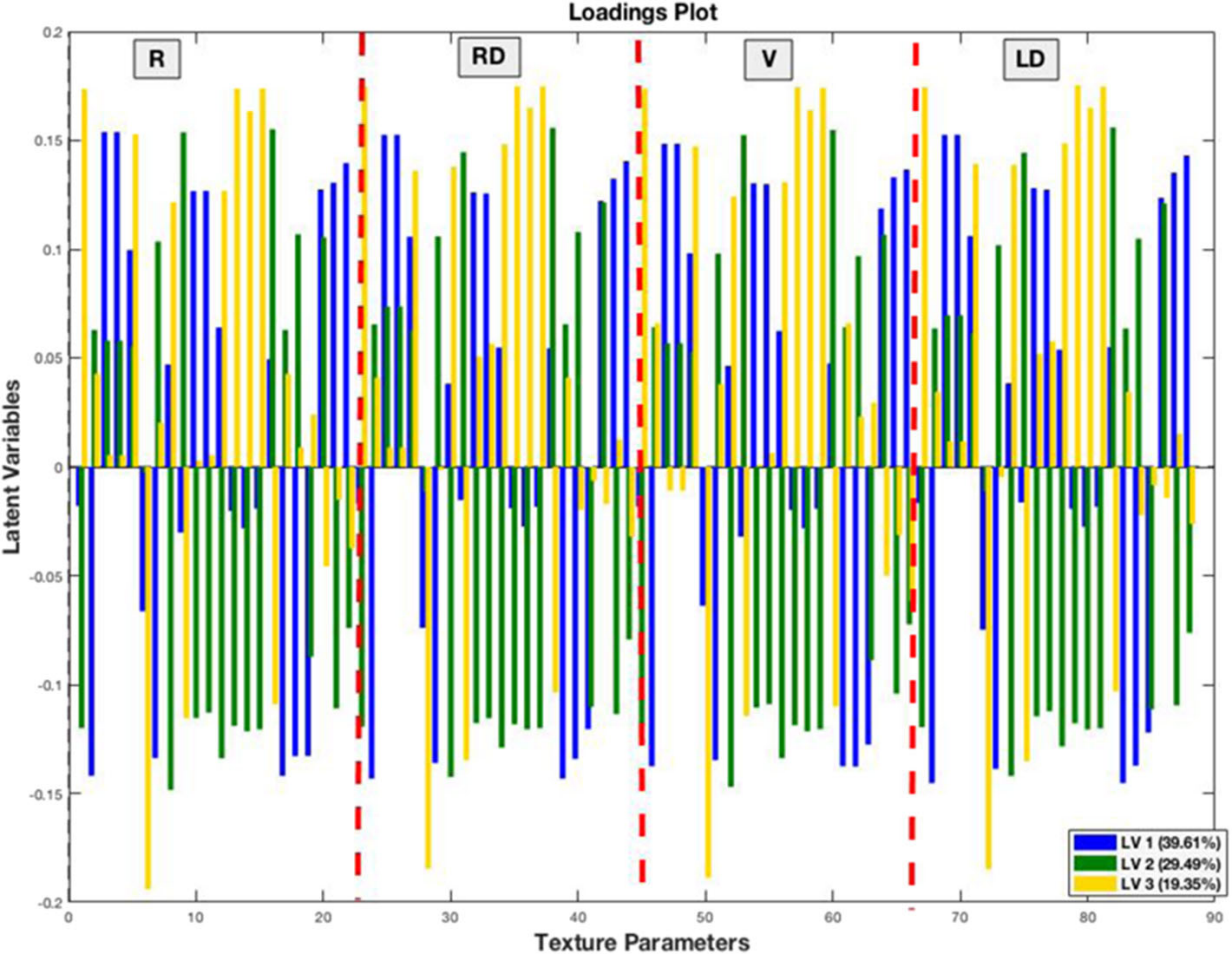
Loadings plot from PLSDA. The loadings plot of the 22 statistical parameters in four orientations (*R* = 0^o^, RD = 45^o^,V = 90^o^,LD = 135^o^) for the first three Latent Variables. This plot shows the relationship of the various texture features that were extracted from GLCM and the percentage of the variation explained in the first three Latent Variables. (Nomenclature for these features is given in Table 1)

By a closer look in the loadings plot of the PLS-DA in Fig. 5 it can be seen that the textural parameters in every of the four orientations provide similar results by explaining the same percentage of variance (LV1 = 39.61%,LV2 = 29.49%,LV3 = 19.35%) in the first three Latent Variables. Therefore a variable selection technique using only one of the four orientations e.g. the horizontal (*R* = 0^O^) could be chosen for further investigation of the texture of the images [23].

Since there is an overlap between the CTPA images of the two diseased groups a further investigation took place in order to determine how the textural parameters differ between these two groups. Usually this is being done with the use of a t-test, which tests the hypothesis that two samples come from the same distribution based on the differences between the means of the samples. T-tests assume normal distributions and are most commonly used when comparing equal sized samples. In the present study the groups had unequal sizes of samples (diseased with PTE (*n* = 7) and diseased without PTE (*n* = 22)). One way to deal with this problem is to use the bootstrap method for hypothesis testing which is a computer-intensive resampling method [24]. The hypothesis tested here is, if the means for seven textural parameters in the horizontal orientation from the group of diseased dogs with PTE are different compared to the textural parameters from the images from the group of diseased dogs without PTE. These seven parameters (entropy, energy, correlation, homogeneity, sum of variance and inverse difference normalized) were chosen because they were considered most relevant for the present analysis [25]. The results showed that the means for six of the seven texture parameters were significantly different between the two diseased groups with only entropy being similar between the two groups.

## Discussion

The objective of the present study was to investigate the performance of quantitative texture analyses of pulmonary parenchyma using co-occurrence matrices with the hypothesis that dogs with macroscopically visible PTE on CTPA would be separable from healthy dogs and from diseased dogs without evidence of PTE.

The results showed that healthy and diseased dogs were readily distinguished using PCA, PLS-DA and SVM on the quantitative data generated using grey level co-occurrence matrices, and the PLS-DA and SVM models generated yielded excellent sensitivities and specificities for classification as healthy.

If PTE only is a localized event occurring inside the vessels, the parenchymateous texture features of dogs with PTE should have been similar to the healthy dogs, which was not the case. Our results suggest that diffuse parenchymal texture changes do occur. DECT studies investigating perfusion changes in rabbits with pulmonary thromboembolism have supported the view that PTE affects the pulmonary parenchyma [26, 27]. Such changes may not be visible to the radiologist and therefore computer assisted analysis like those used in this study will likely add value to the image interpretation, when it is not possible to perform DECT. Detection of such previously unappreciated textural changes could have clinical importance to the patient since widespread parenchymal changes can have serious consequences on for example right sided heart function [28].

The parenchymatous changes found in the dogs with PTE in the present study may be due to locally embedded micro thrombi or merely hypoxia in the parenchyma due to a larger up-stream obstruction of a vessel. It can be argued that dogs may be less prone than humans to large (single) clot PTE obstruction. In humans emboli often arise from Deep Vein Thrombosis (DVT) of the lower limbs. DVT has not been described in dogs yet, although dogs can develop venous thrombosis elsewhere in the body such as vena cava, splenic or portal veins. PTE in dogs can occur in conjunction with a vast number of different diseases and syndromes, but a primary thrombus at other sites than the lungs is often either not present or not reported [29–32]. Histopathology would have been required to assess if the parenchymateous changes in the dogs with PTE in this study were due to (micro) thrombi.

The poorer performance in the texture based classifications of diseased dogs with PTE and diseased dogs without PTE in PLS-DA and SVM may be due to the fact that some of the dogs in both groups had diseases known to affect the pulmonary parenchyma including pulmonary fibrosis, pneumonia or various sizes of pulmonary masses. However, performing the bootstrap method, the results showed that means from 6 out of 7 texture parameters were significantly different with only entropy being similar between these two groups. This could be an indication that the texture is different between the two diseased groups and that the bad prediction in PLS-DA and SVM occurred due to the small sample sizes with in the groups [33].

The present study has some limitations. First of all, since these were clinical patients as indicated above, post mortem examinations and histopathology was not available so the exact health status of each diseased dog with regard to small peripheral or micro thrombi and the true nature and extent of possible additional lung disease was not known. Second, the groups were divided on the basis of a radiological evaluation, which cannot extend to small subsegmental thrombi or micro thrombi and so may be insensitive to parenchymateous texture changes identified by GLCM texture analysis used in this study. Machine-learning techniques such as those used in this paper are especially sensitive to cases assigned to the wrong disease group, as this causes generation of a model based on flawed assumptions. Finally, supervised and unsupervised classification models require larger datasets than available in this study and the number of dogs within each group should ideally have been similar.

Despite the limitations, the very clear division between scans from healthy and diseased dogs indicate that quantitative texture analysis using GLCM can be used to discriminate healthy and abnormal pulmonary parenchyma. Therefore, the findings of the present study justify continued on-going efforts in the development of CAD techniques.

## Conclusion

Though it was not possible to adequately distinguish dogs with PTE from other diseased dogs with pulmonary pathology, the present study exemplifies a new approach to radiological diagnosis combining traditional image reading methods with statistical analyses of large amounts of image data. The usefulness of the latter may be to act as a ‘second reader’ for the radiologist, flagging up abnormal patients that require additional attention. Specifically, the results indicate that texture analysis based on grey level co-occurrence matrices is an effective tool for distinguishing healthy from abnormal lung tissue based on CTPA images.

## Additional file

Additional file 1: Elaboration on statistical methods. (DOCX 99 kb)

## Abbreviations

CAD: Computer assisted diagnosis
CT: Computed tomography
CTPA: Computed tomography pulmonary angiography
DECT: Dual energy computed tomography
DICOM: Digital imaging and communications in medicine
DVT: Deep vein thrombosis
GLCM: Grey level co-occurrence matrix
HU: Hounsfield unit
PCA: Principal component analysis
PLS-DA: Partial least squares discriminant analysis
PTE: Pulmonary thromboembolism
ROC: Receiver operator characteristics
SVM: Support vectors machine
TIFF: Tagged image file format
