# Supplementary material for: Texture analysis of pulmonary parenchymateous changes related to pulmonary thromboembolism in dogs – a novel approach using quantitative methods
Source: BMC Vet Res. 2017 Jul 11;13:219. doi: 10.1186/s12917-017-1117-1 (PMC5505049; doi:10.1186/s12917-017-1117-1)
Supplement: Additional file 1: — Elaboration on statistical methods. (DOCX 99 kb) [file 12917_2017_1117_MOESM1_ESM.docx]

Supplementary Material, BVET-D-15-00263

1. Sensitivity and Specificity Ratio Calculations

The TP (True Positive), FN (False Negative), TN (True Negative), FN (False Negative) and ROC curves for multiclass problems are being calculated based on Pérez NF et al 2009.

Since we have three classes Y1= Healthy, Y2=diseased with PTE, Y3= diseased without PTE "the one versus the rest strategy" is being used (Milgram J et al 2006). This means that the TP for the Healthy class is simply all the Y1 instances (here the CTPA images) that are classified as Healthy. The TN are all Non Y1 instances (meaning Y2 and Y3 together) that are NOT classified as Healthy. FP for Y1 class are all Non Y1 instances that classified as Healthy and FN of Y1 are all Y1 instances that are NOT classified as Y1. The same procedure applies for the other two classes. As an example Supplementary Table 1 is the confusion table output from the cross-validation predictions from PLS-DA. The TP, FN, TN and FN for the healthy class will have values based on "the one versus the rest strategy" and they will be equal to TP = 746, FP = 42, FN = 44, TN = 1052. Sensitivities and specificities can then be calculated as shown in Supplementary Table 2.

Supplementary Table 1.Confusion table from the cross-validation results from PLSDA predictions

| Confusion Table of Cross-Validation of PLS-DA Predictions  Actual Class | | | |
| --- | --- | --- | --- |
|  | Healthy | With PTE | Without PTE |
| Predicted as Healthy | 746 | 10 | 32 |
| Predicted as diseased with PTE | 17 | 90 | 296 |
| Predicted as diseased without PTE | 27 | 196 | 470 |

Supplementary Table 2. Sensitivity and Specificity ratios for the Healthy Class

| HEALTHY CLASS | Prediction Condition is positive | | Prediction Condition is negative |
| --- | --- | --- | --- |
| Test is positive | TP = 746 | | FP =10+32 = 42 |
| Test is negative | FN = 17+27 = 44 | | TN = 90+296 +196+ 470 = 1052 |
| Sensitivity Ratio of the Healthy Class | | ${Sensitivity}_{HEALTHY}= \frac{TP}{(TP+FN)}=\frac{746}{746+44}= 0.94430$ | |
| Specificity Ratio of the Healthy Class | | ${Specificity}_{HEALTHY}= \frac{TN}{(FP+TN)}=\frac{1052}{42+1052}=0.9616$ | |

1. Bootstrap Hypothesis Testing

**Data from two independent Samples**

Observed Sample 1: CTPA images from dogs diseased with PTE of size n: $\left\{ x_{1},x_{2},\ldots{,x}_{n} \right\}=\mathbf{X}_{n}$

Observed Sample 2: CTPA images from dogs diseased without PTE of size k: $\left\{ x_{1},x_{2},\ldots{,x}_{k} \right\}=\mathbf{X}_{k}$

Observed sample mean difference: $m_{d}= m_{n}-m_{k}$

**Hypothesis and significance level**

$H_{0}:$ Both samples are from the same population $\mu_{d}=0$

$H_{1}:$ Both samples are NOT from the same population $\mu_{d}\neq0$

alpha level: a= 0.025

**Bootstrap Procedure**

1. The two observed samples are merged together (n+k) observations
2. A bootstrap sample of (n+k) observations is being drawn with replacement from the merged sample
3. The means of the first n and k observations are being calculated ($m_{n,},m_{k}$) together with the difference $m_{d}= m_{n}-m_{k}$(1)
4. The steps 2 and 3 are being repeated for B times (in our study 10000 times) and we obtain B values of the mean difference $m_{d}$ (we call it test statistic)
5. Estimate the confidence interval CI for the bootstrap samples
6. Does the CI covers zero? Reject $H_{0}$if zero is not included inside the CI ,retain $H_{0}$ otherwise

References:

Milgram J, Cheriet M, Sabourin R: "One against one" or "One against all": Which One is better for Handwriting Recognition with SVMs?. In Tenth International Workshop on Frontiers in Handwriting Recognition. 2006. Suvisoft

Pérez NF, Ferré J, Boqué R. Calculation of the reliability of classification in discriminant partial least-squares binary classification. Chemom Intell Lab Syst 2009, 95:122-128
